# Influence of Dietary Behaviors on Dyslipidemia in Pregnant Women and Its Effects on Physical Development of Fetuses and Infants: A Bidirectional Cohort Study

**DOI:** 10.3390/nu13103398

**Published:** 2021-09-27

**Authors:** Chenyang Li, Xuening Li, Dan Wu, Qi Chen, Zhe Xiao, Deliang Wen, Lingling Zhai, Lihong Jia

**Affiliations:** 1 Department of Child and Adolescent Health, School of Public Health, China Medical University, Shenyang 110122, China; 18855179727@163.com (C.L.); lixuening1988@163.com (X.L.); wudan@cmu.edu.cn (D.W.); chenqiemmm@163.com (Q.C.); xzcmu2019@163.com (Z.X.); 2 Liaoning Key Laboratory of Obesity and Glucose/Lipid Associated Metabolic Diseases, Shenyang 110122, China; dlwen@cmu.edu.cn; 3 Department of Pediatrics, The Fourth Affiliated Hospital of China Medical University, Shenyang 110032, China; 4 Institute of Health Science, China Medical University, Shenyang 110122, China

**Keywords:** dyslipidemia, dietary, physical development, leptin, pregnancy

## Abstract

Background: Gestational diabetes can alter the trajectory of fetal development, but there are few studies on the effects of abnormal lipid metabolism on physical development of infants. We aimed to explore the prevalence of maternal dyslipidemia, its influencing factors and effects on the physical development of fetuses and infants, as well as the role of leptin in this process. Methods: Questionnaire surveys and main outcome measures were administered among 338 pairs of pregnant women and newborns. Results: The detection rate of maternal dyslipidemia was 31.5%. The median levels of TG (triglyceride) and TG/HDL (high-density lipoprotein) ratio were higher in large-for-gestational-age (LGA) newborns. Birth weight was positively related to infants’ height and weight at six months and one year old (*p <* 0.05). Leptin was positively related to TG levels of pregnant women and newborns’ birth weight (*p <* 0.05). Logistic regression analysis showed that having greater than or equal to four meals a day (OR = 6.552, 95%CI = 1.014–42.338) and liking to eat lightly flavored food during pregnancy (OR = 1.887, 95%CI = 1.048–3.395) were independent risk factors of maternal dyslipidemia. Conclusions: The prevalence of dyslipidemia was relatively high in pregnant women and was affected by dietary behaviors. Abnormal lipid levels during pregnancy could affect weight and length at birth, which might be associated with increasing leptin levels in cord blood, and then the weight of infants would be influenced by birth weight.

## 1. Introduction

The prevalence of obesity in children and adolescents has increased substantially around the world [1]. The causes of childhood obesity are complex. High birth weight has been shown to be a risk for childhood obesity, which is influenced by the early intrauterine environment. The Developmental Origins of Health and Disease (DOHaD) hypothesis suggests that nutrient excess in utero results in an acquired susceptibility to metabolic disease later in life and the early intrauterine environment is closely related to the metabolism of pregnant women [2]. At present, there are more reports about the effect of gestational diabetes on the physical development of fetuses. However, there are fewer studies that have focused on abnormal lipid metabolism of pregnant women, and its effect on physical development of fetuses. It is of great practical significance to study the relationship between lipid metabolism of pregnant women and the physical development of fetuses and infants, because of the higher prevalence of dyslipidemia in adulthood in China now. The metabolic pathways underlying early developmental programming in human infants at risk for obesity remain poorly understand. Studies indicate that leptin could affect glucose and lipid metabolism by direct or indirect approaches, through central mechanisms and via metabolically active hormones, such as insulin [3,4,5]. The aim of this study was to explore the effect of blood lipid levels of pregnant women on leptin levels in cord blood, and physical development of fetuses and infants.

The changes in diet and exercise behavior, and the occurrence of complex physiological phenomena during pregnancy, will affect the levels of maternal blood lipid, including total cholesterol (TC), triglyceride, low-density lipoprotein (LDL) and high-density lipoprotein cholesterol. Elevation of blood lipid levels due to pregnancy to a certain extent is physiological [6]. Nevertheless, when the blood lipid levels surpass the physiological range and develop into dyslipidemia (DLP), adverse pregnancy outcomes such as preeclampsia, prolonged pregnancy, preterm birth and macrosomia will occur [7,8,9]. Current researches’ conclusions about the influencing factors of maternal dyslipidemia are not consistent, so further study is needed to settle targeted preventive measures in the future to reduce the occurrence of maternal dyslipidemia and the incidence of adverse pregnancy outcomes.

The objective of the present study was to explore the influencing factors of women’s dyslipidemia during late gestation and its effects on the physical development of fetuses and infants, as well as the role of leptin in this process, in order to prevent maternal dyslipidemia and ensure the healthy growth and development of infants and young children.

## 2. Materials and Methods

### 2.1. Research Objects

A population-based bidirectional cohort study of 338 pairs of pregnant women and newborns was performed between January 2020 and July 2021. The participants in this study were recruited from a third-class A hospital by convenient sampling in Shenyang, China. This study was approved by the Medical Ethics Committee of the Fourth Affiliated Hospital of China Medical University and all participants had provided written informed consent (Reference number: EC-2019-KS-027).

Subjects were included in the study if: (1) they were aged ≥18 years old; (2) they were natural singleton conception; (3) the newborns were live birth. Subjects were excluded from the study if: (1) they were multiple pregnancy; (2) the newborns were birth defects and stillbirth; (3) they suffered from acute and chronic infectious diseases, tumors, cardiovascular diseases or diabetes before pregnancy; (4) they had mental and intellectual disabilities.

### 2.2. Research Methods

The basic information of pregnant women including general demographic characteristics, dietary frequency, dietary habits, exercise and sleep conditions was gathered by the trained pediatricians through face-to-face surveys using questionnaires, while the standard height and weight measuring instrument was used to measure the height and weight of pregnant women by professionals. Calculated body mass index (BMI) was based on these parameters: BMI = weight (Kg)/height^2^ (m)^2^.

Diet-related information included the following aspects: (1) Main food intake frequencies included grains, meats, milk products, soy products, seafoods, vegetables, fruits, etc. (2) Snack intake frequencies included canned food, fried food, nuts, sweets, puffed food, beverages, etc. (3) Eating habits included breakfast, often to eat food before going to bed, whether to eat on time, meals per day, taste, types of drinking water, etc. Definition of snack intake frequencies was as follows: 4–5 times per week was regularly, 2–3 times per week was occasionally, and ≤1 time per week was barely.

Fasting serum lipids: We informed pregnant women to fast for 12 h before getting up in the morning for blood sampling. TC, TG, HDL and LDL levels were detected by a fully automated biochemical analyzer (Siemens ADVIA2400). The abnormal lipid levels were evaluated by the Williams Obstetrics when TC, TG and LDL were, respectively, higher than 7.73 mmol/L, 4.31 mmol/L and 4.76 mmol/L, and HDL was lower than 1.34 mmol/L [10]. Pregnant women were diagnosed with dyslipidemia if any one of the above four blood lipids was abnormal. Pregnant women with dyslipidemia were divided into the case group, and the others were divided into the control group.

The birth weight (kg) and birth length (cm) of newborns were gathered from delivery records, and then the height (cm) and weight (kg) of infants at six months and one year old were followed up by the trained pediatricians. We divided newborns into large-for-gestational-age and appropriate for gestational age according to the growth reference standards of newborns’ birth weight of different gestational ages in China. Large-for-gestational-age infants were identified when birth weight was >90th percentile standardized birth weight [11].

Cord blood samples obtained from umbilical veins were processed within one hour after birth. Serum was separated by centrifugation and stored at −80 °C until assayed. Leptin in 57 random neonatal cord blood samples was detected by ELISA kit (R&D Systems, Inc., Shanghai, China).

### 2.3. Statistical Analysis

Data management and analysis were performed using SPSS 22.0. Categorical variables were expressed as n (%), chi-squared test or fisher exact test was used to compare differences between groups. Normality tests for continuous variables were conducted. Non-normality variables were expressed as median (quartiles), and the Mann–Whitney U test was used to compare differences between groups. Correlation between two continuous variables was tested by Partial correlation. The logistic regression analysis was used to assess the risk factors of maternal dyslipidemia. The significance level of the *p* value was set at 0.05.

## 3. Results

A total of 338 pairs of pregnant women and infants participated in this study. The age (29.31 ± 3.99 years old), height (162.30 ± 5.33 cm), weight before pregnancy (58.31 ± 9.99 kg) and antenatal weight (74.75 ± 10.54 kg) of pregnant women, the birth weight (3.38 ± 0.41 kg) and birth length (50.58 ± 1.57 cm) of newborns, the weight (8.75 ± 1.07 kg) and height (69.34 ± 2.77 cm) of infants at six months, and the weight (10.36 ± 1.15 kg) and height (77.10 ± 2.70 cm) of infants at one year old are shown in Table 1.

According to the Williams Obstetrics, the overall abnormal rates of TC, TG, HDL-c, LDL-c of pregnant women were 7.1%, 23.4%, 1.7%, 6.4%. The prevalence of dyslipidemia was 31.5% under the standards that one of the above was abnormal. Meanwhile, the prevalence rate of maternal dyslipidemia was 5.2% as per self-report, far lower than 31.5% (*p <* 0.001), as shown in Table 2.

The median levels of TG and TG/HDL ratio were higher in LGA newborns; infants’ height and weight at six months and one year old were higher in LGA newborns than in appropriate for gestational age newborns, shown in Table 3.

The HDL levels were negatively related to the weight and height of infants at six months in the case of controlling birth weight and birth length (r_s_ = −0.157, *p* = 0.017; r_s_ = −0.144, *p* = 0.029, respectively), while other blood lipid levels had nothing to do with the weight and height of infants in the case of controlling birth weight and birth length (*p* > 0.05). Birth weight was positively related to infants’ height and weight at six months and one year old (r_s_ = 0.322, *p <* 0.001; r_s_ = 0.344, *p <* 0.001; r_s_ = 290, *p <* 0.001; r_s_ = 0.316, *p <* 0.001, respectively), shown in Table 4.

**Figure 1:**
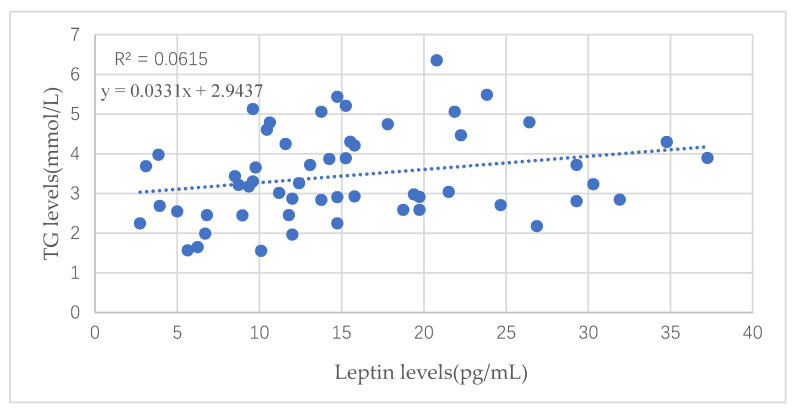
Correlation between leptin in cord blood and TG levels of pregnant women.

There was a positive correlation between leptin in cord blood and TG levels of pregnant women in the third trimester of pregnancy (*p* = 0.025), shown in Figure 1, while the leptin had nothing to do with TC levels, HDL-c levels, LDL-c levels or the TC/HDL ratio, TG/HDL ratio, or LDL/HDL ratio (*p* > 0.05, not shown in figures below).

**Figure 2:**
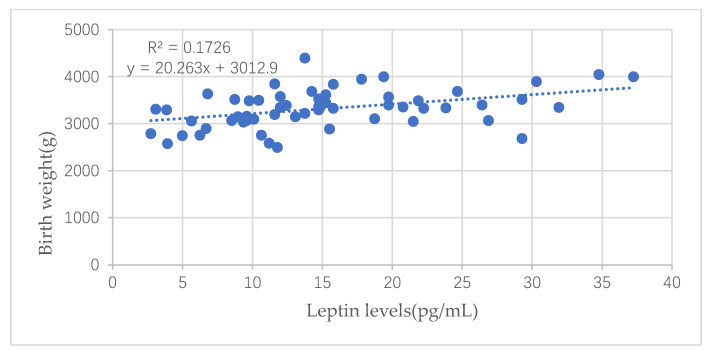
Correlation between leptin in cord blood and the birth weight.

Leptin in cord blood was positively related to newborns’ birth weight (*p <* 0.001), shown in Figure 2, but was not directly related to infants’ weight at six months and one year old (*p* > 0.05, not shown in figures below).

Compared with the control group, the proportion of pregnant women whose age was higher than 30 years old, family financial status was well, or weight gain was excessive during pregnancy was higher in the case group (44.1% vs. 38.1%; 10.8% vs. 5.4%; 38.7% vs. 33.2%, respectively), and the proportion of passive smoking before or after pregnancy was lower in the case group (41.9% vs. 46.0%; 28.0% vs. 32.7%, respectively). None of the differences above were statistically significant (*p* > 0.05). There was no significant difference in the proportion of pregnant women with family history of diabetes or hypertension between the control group and the case group (*p* > 0.05), shown in Table 5.

Compared with the control group, the intake frequencies of common food, such as coarse grain, vegetables, fruits, soy products, and milk products, greater than or equal to 1 time/day for pregnant women during pregnancy were a bit higher, while the intake frequencies of lean meat and eggs greater than or equal to 1 time/day and of seafoods and liver greater than or equal to 4 times/week were a bit lower in the case group. None of the differences above were statistically significant (*p* > 0.05), as shown in Table 6.

The ‘barely intake’ frequencies of canned food, barbecue, fried food, puffed food, beverages and coffee of pregnant women during pregnancy between the control and case groups were quite unanimous; all of them were higher than 85% (*p >* 0.05). The ‘barely intake’ frequencies of sweets and the ‘occasionally or regularly intake’ frequencies of nuts were around 60% in both control and case groups (*p >* 0.05). The ‘regularly intake’ frequencies of health products and the proportion of vitamin D supplement were between 71% and 81% in both control and case groups (*p >* 0.05), as shown in Table 7.

Compared with the control group, the proportion of pregnant women who had equal to or more than three meals a day or who liked to eat lightly flavored food during pregnancy was higher in the case group (46.3% vs. 28.2%; 78.5% vs. 65.8%, *p <* 0.05). There were no statistically significant differences in eating habits including breakfast, often eating food before going to bed, eating on time, other tastes, and types of drinking water between the control and case groups, as shown in Table 8.

Compared with the control group, the proportion of pregnant women who had an elevator at home, watched TV more than 2 h per day, and played mobile more than 3 h per day were higher in the case group. None of the differences above were statistically significant (*p >* 0.05). Furthermore, ways to go to work, exercise time per day, liking exercise before or after pregnancy, and sleep conditions between the control and case groups were about the same (*p <* 0.05), as shown in Table 9.

Variables with a *p* value less than 0.05 in the univariate analysis were included in the logistic regression analysis. The result showed that having greater than or equal to four meals a day (OR = 6.552, 95%CI = 1.014–42.338) and liking to eat lightly flavored food during pregnancy (OR = 1.887, 95%CI = 1.048–3.395) were independent risk factors of maternal dyslipidemia, as shown in Table 10.

## 4. Discussion

We found the prevalence rate of maternal dyslipidemia was 31.5% according to the Williams Obstetrics. A previous study showed that the detection rates of maternal dyslipidemia varies greatly by different criteria [12]. This was consistent with our findings as we found the prevalence rate of maternal dyslipidemia was 5.2% by self-report, obviously lower than 31.5%. We concluded that current society often ignores abnormal blood lipid levels during pregnancy as the self-report rate of dyslipidemia was obviously lower. This phenomenon should arouse our attention and health education in this field should be strengthened from now on.

We found birth weight was affected by abnormal TG levels of pregnant women, which agreed with the results of many studies [13,14,15]. In our study, the median levels of TG/HDL ratio were higher in LGA newborns, which agreed with the study that found the TG/HDL ratio showed an obvious dose-dependent relationship with the occurrence of macrosomia [16]. Consistent with our result, most studies had not found relationships between maternal serum TC and LDL levels and birth weight [13,16]. Weight in early life was highly associated with weight and quality of life in children, adolescents and adults [17,18,19]. We concluded that TG levels in the third trimester of pregnancy could influence infants’ height and weight indirectly through the influence of birth weight, as the birth weight was positively related to infants’ height and weight at six months and one year old. The number of adipocytes would no longer decrease once they were formed, leading to obesity in children at a younger age, which could represent a hidden danger of being overweight or obese in the future [20]. Therefore, blood lipid levels of pregnant women should be as much a concern during pregnancy as blood sugar levels.

Leptin could lead to an increase in triglyceride hydrolysis and a suppression of lipogenesis and in vivo studies have obviously demonstrated that leptin can reduce body fat mass [21]. Our study showed that leptin in cord blood was positively correlated with the maternal serum TG levels in the third trimester of pregnancy and birth weight of fetuses, but was not directly related to infants’ weight at six months and one year old, consistent with Hauguel-de Mouzon, S.’s findings [22]. This indicates that high TG levels in the third trimester of pregnancy would promote the synthesis of leptin in mothers, and then it would be transmitted into the umbilical cord blood, which would eventually have a direct impact on the growth and development of fetuses. Later, the leptin in the cord blood would indirectly affect the physical development of infants after birth as birth weight was positively related to infants’ height and weight at six months and one year old. The formation of leptin receptors in fetuses could be influenced by the leptin in cord blood, therefore, the leptin receptor gene was considered a biological pathway related to childhood obesity development [23,24].

The influencing factors of gestational dyslipidemia in the third trimester of pregnancy were explored. Studies have reported that the maternal lipid levels were affected by maternal age, BMI before pregnancy and weight gain during pregnancy [25,26,27]. However, these relationships were not found in our study. Therefore, the baseline information of the two groups of pregnant women was consistent. A nutrient-rich maternal diet during pregnancy was essential to pregnant womens’ health and fetuses’ development [28]. However, diets with high sugar, high saturated fat and an unreasonable proportion of processed food might increase the risk of adverse pregnancy outcomes [29,30,31]. Interestingly, the results of this study showed no relationship between maternal dyslipidemia and the food intake frequencies or the habit of eating and drinking a lot. We could see the ‘barely intake’ frequencies of relatively unhealthy food, such as canned food, barbecue, fried food, puffed food, beverages and coffee, were higher than 85% in both control and case groups of pregnant women. The dietary habits of pregnant women were healthy while they tended to eat breakfast regularly, eat meals on time, drink boiled water other than sweet drinks, and so on. The vigorous propaganda about the hazards of high sugar, high saturated fat and processed food and the self-protection awareness of pregnant women might explain this phenomenon. Meanwhile, there were no statistically significant differences in exercise and sleep conditions of pregnant women between the control and case groups, however, we still found they tended to spend more time on TV and phones and less time doing exercise on the whole. As exercise and sleep conditions could influence blood lipid levels, this phenomenon should arouse our attention [32,33].

Logistic regression analysis showed that having greater than or equal to four meals a day and liking to eat lightly flavored food during pregnancy were independent risk factors for maternal dyslipidemia in the third trimester of pregnancy. Although liking to eat lightly flavored food during pregnancy was good for health, it might lead to a lack of protein and excessive carbohydrate intake. If the energy exceeded the body’s needs, carbohydrate-decomposed sugar would be converted into fat in the liver, leading to increased blood lipid levels in pregnant women [34,35]. Our results showed that the intake frequency of staple foods, vegetables and fruits of at least once a day reached around 80%, while the intake frequency of lean meat, eggs and milk products of at least once a day was only about 50%. Furthermore, the intake frequencies of seafoods and liver were not enough either. The digestive system would stimulate the body to eat more carbohydrates or increase the number of meals if the intake of protein and fat was insufficient, as they could bring a feeling of fullness and prolong the emptying time of the stomach [36,37,38]. Eating a light diet may reduce satiety, and lead to an increase in the number of meals and excessive intake of carbohydrates, which will eventually lead to maternal dyslipidemia. Thus, we should strengthen the guidance of dietary balance during pregnancy to prevent the occurrence of maternal dyslipidemia in the future.

Our study indicated that there was a positive relationship of maternal dyslipidemia during late gestation and the birth index and infants’ development as it was a population-based bidirectional cohort study. However, the follow-up bias was inevitable. Our research showed that the number of meals per day and preferring lightly flavored food during pregnancy might be risk factors for maternal dyslipidemia. Another limitation of our investigation was that the intake of various foods during pregnancy was not quantified, and it was impossible to compare the energy supply proportions of the three major macronutrients.

## 5. Conclusions

Transient mild dyslipidemia during pregnancy could steadily return to normal after delivery, while more serious dyslipidemia was difficult to correct [39]. Dyslipidemia in pregnant women could affect birth weight by increasing leptin levels in cord blood, and then influencing the physical development of young children. Thus, we should strengthen the monitoring of blood lipid levels of women during pregnancy. The habits of diet during pregnancy were basically reasonable under vigorous publicity, but the implementation of dietary balance still needed more guidance.

## Figures and Tables

**Table 1:** The basic information of pregnant women and the height and weight of newborns and infants.

Items	N	M ± SD/%
Pregnant women		
Age (y)	338	29.31 ± 3.99
Height (cm)	338	162.30 ± 5.33
Weight before pregnancy(kg)	338	58.31 ± 9.99
Antenatal weight (kg)	338	74.75 ± 10.54
Newborns		
Birth weight (kg)	329	3.38 ± 0.41
Birth length (cm)	329	50.58 ± 1.57
Six months		
Weight (kg)	315	8.75 ± 1.07
Height (cm)	315	69.34 ± 2.77
One year old		
Weight (kg)	280	10.36 ± 1.15
Height (cm)	280	77.10 ± 2.70

Note: Some of the variables had missing values.

**Table 2:** The detection rates of abnormal blood lipids of pregnant women.

Items	N	Abnormal	Detection Rate
TC (mmol/L)	295	21	7.1%
TG (mmol/L)	295	69	23.4%
HDL-c (mmol/L)	295	5	1.7%
LDL-c (mmol/L)	295	19	6.4%
One of the above was abnormal	295	93	31.5%
Self-report	291	15	5.2%

Note: Some of the variables had missing values.

**Table 3:** Comparison of blood lipid levels of pregnant women and the weight and height of infants in LGA and appropriate for gestational age groups (median, quartiles).

Items	LGA (*n* = 71)	Appropriate for Gestational Age (*n* = 230)	Test Value	*p* Value
TC(mmol/L)	6.04(5.39, 7.01)	6.01(5.35, 6.86)	0.166	0.868
TG(mmol/L)	3.90(2.94, 4.68)	3.26(2.69, 4.07)	2.668	0.008
HDL(mmol/L)	1.87(1.66, 2.11)	1.90(1.65, 2.24)	0.587	0.558
LDL(mmol/L)	3.07(2.42, 3.72)	3.13(2.52, 3.95)	0.933	0.351
TC/HDL	3.19(2.79, 3.57)	3.07(2.77, 3.59)	0.493	0.622
TG/HDL	2.08(1.59, 2.68)	1.74(1.35, 2.20)	2.746	0.006
LDL/HDL	1.65(1.23, 1.94)	1.62(1.33, 2.05)	0.686	0.493
Weight (six months, kg)	9.00(8.50, 9.60)	8.50(8.00, 9.50)	2.973	0.003
Height (six months, cm)	70.00(69.00, 72.00)	69.00(67.50, 70.50)	3.743	<0.001
Weight (one year old, kg)	10.50(10.00, 11.50)	10.00(9.50, 11.00)	3.128	0.002
Height (one year old, cm)	78.50(76.00, 80.00)	76.50(75.00, 78.50)	3.739	<0.001

**Table 4:** Correlation between blood lipid levels of pregnant women, birth weight and the height and weight of infants.

Items	Weight (Six Months, kg)	Height (Six Months, cm)	Weight (One Year Old, kg)	Height (One Year Old, cm)
	rs (*n* = 229)	rs (*n* = 229)	rs (*n* = 229)	rs (*n* = 229)
TC (mmol/L)	0.003	0.008	−0.015	−0.025
*p* value	0.966	0.901	0.815	0.701
TG (mmol/L)	0.073	0.014	0.018	0.001
*p* value	0.272	0.835	0.780	0.990
HDL (mmol/L)	−0.157	−0.144	−0.053	−0.023
*p* value	0.017	0.029	0.421	0.733
LDL (mmol/L)	0.013	0.038	0.006	0.005
*p* value	0.845	0.571	0.926	0.942
TC/HDL	0.100	0.102	0.017	−0.005
*p* value	0.131	0.123	0.799	0.937
TG/HDL	0.077	0.025	0.010	−0.004
*p* value	0.247	0.704	0.884	0.957
LDL/HDL	0.092	0.124	0.041	0.031
*p* value	0.162	0.061	0.537	0.644
Birth weight (kg)	0.322	0.344	0.290	0.316
*p* value(*n* = 251)	<0.001	<0.001	<0.001	<0.001

**Table 5:** Comparison of the basic situation of pregnant women in the control and case groups (*n*, %).

Items		Control Group (*n* = 202)	Case Group (*n* = 93)	Test Value	*p* Value
Age	≤24	13(6.4)	8(8.6)	2.388	0.496
	25–29	109(54.0)	43(46.2)		
	30–35	59(29.2)	34(36.6)		
	>35	18(8.9)	7(7.5)		
Education	high school or below	44(21.8)	25(26.9)	0.820	0.664
	Undergraduate	140(69.3)	61(65.6)		
	Master degree or above	13(6.4)	6(6.5)		
Family monthly income (yuan)	>3000	178(88.1)	83(89.2)	0.570	0.450
	≤3000	12(5.9)	8(8.6)		
Family financial status	Well	11(5.4)	10(10.8)	2.662	0.264
	Normal	156(77.2)	69(74.2)		
	Bad	31(15.3)	13(14.0)		
BMI before pregnancy	Thin	33(16.3)	14(15.1)	0.708	0.871
	Normal weight	117(57.9)	54(58.1)		
	Overweight	34(16.8)	16(17.2)		
	Obesity	12(5.9)	8(8.6)		
Weight gain(kg)	Insufficient	50(24.8)	19(20.4)	1.045	0.593
	Suitable	79(39.1)	37(39.8)		
	Excessive	67(33.2)	36(38.7)		
Passive smoking before pregnancy	Yes	93(46.0)	39(41.9)	0.425	0.514
	No	105(52.0)	52(55.9)		
Passive smoking after pregnancy	Yes	66(32.7)	26(28.0)	0.788	0.375
	No	127(62.9)	64(68.8)		
					
Family history of diabetes	Yes	25(12.4)	14(15.1)	0.340	0.560
	No	174(86.1)	79(84.9)		
Family history of hypertension	Yes	26(12.9)	15(16.1)	0.470	0.493
	No	172(85.1)	78(83.9)		
Mood during pregnancy	Well	78(38.6)	32(34.4)	0.417	0.812
	Better	92(45.5)	45(48.4)		
	Normal	30(14.9)	13(14.0)		

Note: Some of the variables had missing values.

**Table 6:** Comparison of the common food intake frequencies of women during pregnancy between the control and case groups (*n*, %).

Items		Control Group (*n* = 202)	Case Group (*n* = 93)	Test Value	*p* Value
Staple food (rice/steamed bun/noodles, etc.)	≥1 time/day	159(78.7)	73(78.5)	0.020	0.887
	<1 time/day	41(20.3)	18(19.4)		
Coarse grain	≥1 time/day	25(12.4)	15(16.1)	0.749	0.387
	<1 time/day	172(85.1)	76(81.7)		
Vegetables	≥1 time/day	166(82.2)	81(87.1)	0.523	0.469
	<1 time/day	32(16.3)	12(12.9)		
Fruits	≥1 time/day	173(85.6)	84(90.3)	0.393	0.531
	<1 time/day	24(11.9)	9(9.7)		
Lean meat (livestock, poultry)	≥1 time/day	91(45.0)	40(43.0)	0.189	0.664
	<1 time/day	108(53.5)	53(57.0)		
Eggs	≥1 time/day	102(50.5)	42(45.2)	1.022	0.312
	<1 time/day	96(47.5)	51(54.8)		
Soy products	≥1 time/day	30(14.9)	15(16.1)	0.074	0.785
	<1 time/day	167(82.7)	76(81.7)		
Milk products	≥1 time/day	102(50.5)	51(54.8)	0.513	0.474
	<1 time/day	96(47.5)	40(43.0)		
Seafoods	≤1 time/week	136(67.3)	68(73.1)	0.748	0.688
	2–3 times/week	42(20.8)	17(18.3)		
	≥4 times/week	19(9.4)	7(7.5)		
Liver	≤1 time/week	161(79.7)	81(87.1)	1.570	0.456
	2–3 times/week	24(11.9)	9(9.7)		
	≥4 times/week	12(5.9)	3(3.2)		

Note: Some of the variables had missing values.

**Table 7:** Comparison of the snack intake frequencies of women during pregnancy between the control and case groups (*n*, %).

Items		Control Group (*n* = 202)	Case Group (*n* = 93)	Test Value	*p* Value
Sausages and cooked meat products	barely	158(78.2)	70(75.3)	0.689	0.708
	occasionally	31(15.3)	18(19.4)		
	regularly	8(4.0)	4(4.3)		
Canned food (pork, beef, fish, mutton, etc.)	barely	196(97.0)	91(97.8)	*	0.554
	occasionally	3(1.5)	0(0.0)		
Barbecue	barely	194(96.0)	90(96.8)	2.314 *	0.436
	occasionally	2(1.0)	3(3.2)		
	regularly	1(0.5)	0(0.0)		
Fried food	barely	188(93.1)	86(92.5)	0.231 *	1.000
	occasionally	8(4.0)	4(4.3)		
	regularly	4(2.0)	2(2.2)		
Nuts (walnuts, peanuts, melon seeds, etc.)	barely	77(38.1)	39(41.9)	0.757	0.685
	occasionally	50(24.8)	19(20.4)		
	regularly	72(35.6)	34(36.6)		
Sweets (ice cream, candies, biscuits, pastries, etc.)	barely	132(65.3)	55(59.1)	3.031	0.220
	occasionally	34(16.8)	24(25.8)		
	regularly	33(16.3)	14(15.1)		
Puffed food (shrimp crackers, potato chips, etc.)	barely	182(90.1)	82(88.2)	1.327	0.515
	occasionally	12(5.9)	5(5.4)		
	regularly	4(2.0)	4(4.3)		
Beverages (juice, cola, sprite, etc.)	barely	173(85.6)	85(91.4)	3.202	0.202
	occasionally	15(7.4)	5(5.4)		
	regularly	10(5.0)	1(1.1)		
Coffee	barely	194(96.0)	91(97.8)	0.994 *	1.000
	occasionally	1(0.5)	0(0.0)		
	regularly	2(1.0)	0(0.0)		
Health products (calcium, zinc, iron, vitamins, etc.)	barely	36(17.8)	14(15.1)	1.902	0.386
	occasionally	15(7.4)	4(4.3)		
	regularly	144(71.3)	75(80.6)		
Vitamin D supplement	Yes	158(78.2)	66(71.0)	2.082	0.149
	No	41(20.3)	26(28.0)		

Note: Some of the variables had missing values; * Fisher exact test was used.

**Table 8:** Comparison of the eating and drinking habits of women during pregnancy between the control and case groups (*n*, %).

Items		Control Group (*n* = 202)	Case Group (*n* = 93)	Test Value	*p* Value
Breakfast	regularly	185(91.6)	81(87.1)	0.708	0.400
	occasionally	16(7.9)	10(10.8)		
Often to eat food before going to bed	Yes	72(35.6)	34(36.6)	0.023	0.879
	No	130(64.4)	59(63.4)		
whether to eat on time	Yes	175(86.6)	80(86.0)	0.020	0.887
	No	27(13.4)	13(14.0)		
Meals per day	1 time	8(4.0)	2(2.2)	12.738	0.005
	2 times	137(67.8)	48(51.6)		
	3 times	51(25.2)	33(35.5)		
	≥4 times	6(3.0)	10(10.8)		
Like to eat lightly flavored food	Yes	133(65.8)	73(78.5)	4.839	0.028
	No	69(34.2)	20(21.5)		
Like to eat fried food	Yes	24(11.9)	10(10.8)	0.080	0.778
	No	178(88.1)	83(89.2)		
Like to eat salty food	Yes	35(17.3)	19(20.4)	0.410	0.522
	No	167(82.7)	74(79.6)		
Like to eat sour food	Yes	50(24.8)	23(24.7)	0.000	0.997
	No	152(75.2)	70(75.3)		
Like to eat sweet food	Yes	67(33.2)	28(30.1)	0.216	0.642
	No	135(66.8)	64(68.8)		
Like to eat spicy food	Yes	53(26.2)	15(16.1)	3.587	0.058
	No	148(73.3)	77(82.8)		
Drink boiled water regularly	Yes	161(79.7)	73(78.5)	0.057	0.812
	No	41(20.3)	20(21.5)		
Drink juice regularly	Yes	7(3.5)	5(5.4)	*	0.527
	No	195(96.5)	88(94.6)		
Drink sweet drinks regularly	Yes	1(0.5)	3(3.2)	*	0.094
	No	201(99.5)	90(96.8)		
Drink carbonated beverages regularly	Yes	3(1.5)	1(1.1)	*	1.000
	No	199(98.5)	92(98.9)		
Drink plastic barreled water regularly	Yes	33(16.3)	19(20.4)	0.735	0.391
	No	169(83.7)	74(79.6)		
Drink plastic bottled water regularly	Yes	25(12.4)	10(10.8)	0.161	0.689
	No	177(87.6)	83(89.2)		

Note: Some of the variables had missing values; * Fisher exact test was used.

**Table 9:** Comparison of pregnant women’s exercise and sleep conditions between the control and case groups (*n*, %).

Items		Control Group (*n* = 202)	Case Group (*n* = 93)	Test Value	*p* Value
Elevator at home	Yes	106(52.5)	51(54.8)	0.016	0.899
	No	88(43.6)	41(44.1)		
Ways to go to work	By walking	26(12.9)	15(16.1)	0.598	0.439
	By cars	135(66.8)	59(63.4)		
TV time per day (h)	<1	78(38.6)	33(35.5)	2.282	0.319
	1–2	58(28.7)	21(22.6)		
	>2	64(31.7)	37(39.8)		
Phone time per day (h)	<1	8(4.0)	2(2.2)	1.877	0.598
	1–2	32(15.5)	15(16.1)		
	2–3	47(23.3)	17(18.3)		
	>3	114(56.4)	59(63.4)		
Exercise time per day(h)	<0.5	52(25.7)	19(20.4)	2.069	0.355
	0.5–1	93(46.0)	51(54.8)		
	>1	55(27.2)	22(23.7)		
Like to exercise before pregnancy	Yes	56(27.7)	24(25.8)	0.055	0.814
	No	144(71.3)	66(71.0)		
Like to exercise after pregnancy	Yes	68(33.7)	32(34.4)	0.027	0.869
	No	131(64.9)	59(63.4)		
Sleeping time(h)	<8	21(10.4)	9(9.7)	0.088	0.767
	≥8	171(84.7)	83(89.2)		
Sleeping quality	Well	88(43.6)	35(37.6)	1.218	0.749
	Better	64(31.7)	33(35.5)		
	Normal	44(21.8)	23(24.7)		
	Bad	6(3.0)	2(2.2)		

Note: Some of the variables had missing values.

**Table 10:** Logistic regression analysis of the impact factors of maternal dyslipidemia.

Items	*β*	S.E.	Wald	*p* Value	OR(95%CI)
Meals per day					
1 time	Ref.				
2 times	0.290	0.814	0.127	0.721	1.337(0.271–6.591)
3 times	0.882	0.828	1.136	0.287	2.416(0.477–12.238)
≥4 times	1.880	0.952	3.899	0.048	6.552(1.014–42.338)
Like to eat lightly flavored food					
No	Ref.				
Yes	0.635	0.300	4.486	0.034	1.887(1.048–3.395)
Constant	−1.796	0.823	4.768	0.029	0.166

## Data Availability

All datasets generated during and analyzed during the current study are not publicly available but are available from the corresponding author on reasonable request.
